# Genetic causal inference between amblyopia and perinatal factors

**DOI:** 10.1038/s41598-022-22121-3

**Published:** 2022-10-27

**Authors:** Ju-Yeun Lee, Sangjun Lee, Sue K. Park

**Affiliations:** 1 Department of Preventive Medicine, Seoul National University College of Medicine, Seoul, South Korea; 2 Integrated Major in Innovative Medical Science, Seoul National University College of Medicine, Seoul, South Korea; 3 Department of Ophthalmology, Myongji Hospital, Hanyang University College of Medicine, Goyang, South Korea; 4 Cancer Research Institute, Seoul National University, Seoul, South Korea; 5 Department of Biomedicine Science, Seoul National University Graduated School, Seoul, South Korea

**Keywords:** Clinical genetics, Risk factors, Eye diseases

## Abstract

Amblyopia is a common visual disorder that causes significant vision problems globally. Most non-ocular risk factors for amblyopia are closely related to the intrauterine environment, and are strongly influenced by parent-origin effects. Parent-origin perinatal factors may have a direct causal inference on amblyopia development; therefore, we investigated the causal association between perinatal factors and amblyopia risk using a one-sample Mendelian Randomization (MR) with data from the UK Biobank Cohort Data (UKBB). Four distinct MR methods were employed to analyze the association between three perinatal factors (birth weight [BW], maternal smoking, and breastfeeding) and amblyopia risk, based on the summary statistics of genome-wide association studies in the European population. The inverse variance weighting method showed an inverse causal association between BW and amblyopia risk (odds ratio, 0.48 [95% CI, 0.29–0.80]; p = 0.004). Maternal smoking and breastfeeding were not causally associated with amblyopia risk. Our findings provided a possible evidence of a significant genetic causal association between low BW and increased amblyopia risk. This evidence may highlight the potential of BW as a predictive factor for visual maldevelopment and the need for careful management of amblyopia risk in patients with low BW.

## Introduction

Amblyopia is a common visual disorder usually diagnosed during childhood. Its present prevalence is reported to be approximately 0.7–2.9%, which varies between populations, and its global prevalence is predicted to increase by more than two times by 2040^1^. Since visual impairment from amblyopia is lifelong and can be profound if not treated at the proper time, it is becoming a significant vision problem worldwide. From a global health perspective, amblyopia became a major chronic disease, and its related social burden has also been increasing accordingly.

Various predisposing risk factors for amblyopia have been documented, including ocular and non-ocular factors. Common ocular risk factors include refractive error, strabismus, and anisometropia^2–4^. The non-ocular risk factors associated with amblyopia were mainly identified as perinatal factors, such as prematurity, Appearance, Pulse, the Grimace, Activity, and Respiration (APGAR) score, maternal smoking during pregnancy, neonatal intensive care unit hospitalization, and breastfeeding^2^. Several clinical studies have been reported on the ocular risk factors related to amblyopia; however, there are only a few studies have reported non-ocular risk factors. Most non-ocular factors presented in previous studies are closely related to the intrauterine environment; thus, amblyopia is assumed to be strongly influenced by the parent-origin effect. For non-ocular factors subject to systemic and environmental influences, the causality and genetic inference of the occurrence of amblyopia needs to be more clearly demonstrated.

Majority of previous studies on non-ocular factors are observational, and even in a few cohort studies, the effect of some non-ocular factors on amblyopia remains controversial. Since long-term data collection is required and the study designs are usually observational in nature to, there is a clinical limit to evaluating the non-ocular factor causality with amblyopia. A more reliable method is needed to determine significant causality, because conventional observational studies may have limitations, such as confounding and reverse causation.

Mendelian Randomization (MR) has been used to improve causal inference using genetic variants related to modifiable exposures to detect causal associations with outcomes^5^. In terms of study design, the MR technique is similar to randomized controlled trial and can provide more credible evidence, than observational studies, on the causal effect of risk factors on an outcome by overcoming observational design limitations^6^. Using this novel method, we assessed the causal inference of genetic variants on the development of amblyopia in European population using UK biobank (UKBB) data. Herein, we focused on parent-origin perinatal factors obtained from the data; however, the direct causal relationship is not yet clear.

## Results

Workflow for the selection of instrumental variables (IVs) for body weight (BW) based on genome-wide association studies (GWAS) summary statistics from the UKBB is shown in Supplementary Fig. 1. The summary statistics, including p-value, beta, standard error (SE), risk alleles, and risk allele frequencies for the association between single nucleotide polymorphism (SNPs) and BW, maternal smoking, and breastfeeding from UKBB are shown in Supplementary Tables 1, 2 and 3.

We obtained 18 independent SNPs predicting BW with genome-wide significance (p < 1 × 10^–6^) from the GWAS summary statistics as IVs after excluding two SNPs (rs9895335 and rs4977838), because of their potential association with amblyopia (Supplementary Table 4 and Table 1). Considering the downstream effect, the results of not moving these SNPs were also presented in Supplementary Fig. 2. In addition, we selected 39 SNPs associated with maternal smoking and 11 SNPs associated with breast feeding from the GWAS summary statistics as IVs with genome-wide significance.

**Table 1:** Shared selected pleotropic loci for single nucleotide polymorphisms (SNPs) associated with birth weight.

Chr	Pos (bp)	SNP	Function	Gene	Mapped phenotypes/eQTL
1p36.22	11,326,788	rs1074078	Intergenic	*MTOR;UBIAD1*	CKD, ALS, tumor, brain, artery, nerve, skin
1q44	244,460,590	rs74226445	Intergenic	*ZBTB18;C1orf100*	Unknown
22q13.1	39,619,814	rs56031201	UTR3	*PDGFB*	Unknown
2q14.2	120,262,615	rs35991747	Intronic	*SCTR*	Thyroid, pancreas
2q24.1	156,753,946	rs10202061	Intergenic	*KCNJ3;LINC01876*	Unknown
2q24.2	163,444,009	rs1385865	Intronic	*KCNH7*	Unknown
5p14.1	24,913,167	rs9283778	Intergenic	*LINC02239;LINC02228*	Thyroid, prostate testis
5p15.2	13,462,423	rs1348694	Intergenic	*LINC02220;DNAH5*	Unknown
5q14.3	87,857,702	rs116552258	ncRNAintronic	*LINC00461*	BMI, muscle, brain, esophagus
5q31.3	144,172,786	rs3906525	Intergenic	*KCTD16;PRELID2*	Unknown
7q31.31	117,535,278	rs6952555	Intergenic	*CTTNBP2;LSM8*	Fibroblast, lower leg skin
8q21.13	83,474,560	rs111654718	Intergenic	*SNX16;LOC101927141*	Unknown
10p11.22	33,758,280	rs151320013	Intergenic	*NRP1;LINC00838*	Unknown
14q21.2	45,117,631	rs8022105	Intergenic	*LOC105370473;LINC02302*	Unknown
16p13.3	1,600,137	rs2281228	Intronic	*IFT140;TMEM204*	Ciliopathies, artery, nerve, muscle, adipose cell, thyroid, brain
17q25.3	76,255,778	rs7218341	Intergenic	*LOC105371910;LINC01993*	Lung cancer
18q21.2	51,265,020	rs72930125	Intergenic	*LINC01919;MBD2*	Unknown
19p13.3	1,852,494	rs3746038	UTR3	*KLF16*	BMI, MAP

*eQTL* expression quantitative trait loci, *CKD* chronic kidney disease, *ALS* amyotrophic lateral sclerosis, *BMI* body mass index, *MAP* mean arterial pressure.

**Figure 1:**
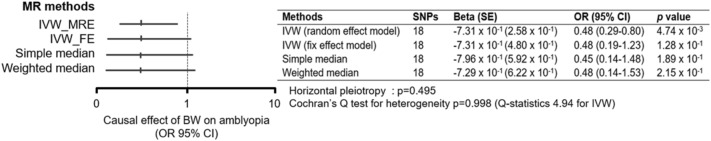
Results of the four main methods for causal inference of birth weight (BW) as risk factors for amblyopia in Mendelian randomization and test for directional horizontal pleiotropy and Cochran’s *Q* test for heterogeneity for BW on amblyopia risk.

**Figure 2:**
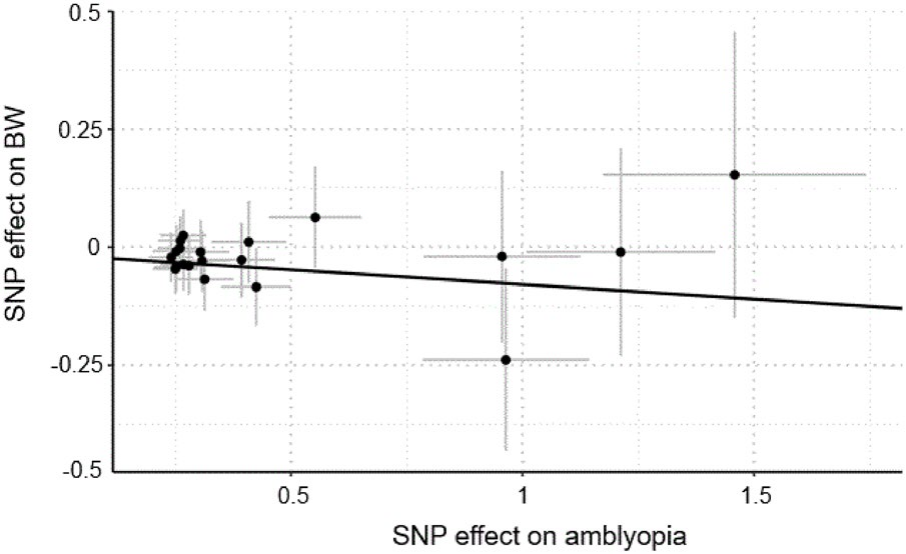
Scatter plots of the estimated effects of single nucleotide polymorphisms (SNPs) on birth weight (BW) against the estimated effects of SNPs on the risk of amblyopia.

Using inverse variance weighting (IVW) with random effect model, we found a possible evidence of a causal inference between BW and amblyopia risk. Genetically predicted BW was negatively associated with amblyopia (beta = − 7.31 × 10^–1^, SE = 2.58 × 10^–1^, p = 4.74 × 10^–3^) (Figs. 1 and 2). Directionally similar associations were estimated using the methods of IVW with fixed effect (beta = − 7.31 × 10^–1^, SE = 4.80 × 10^–1^), simple median (beta = − 7.96 × 10^–1^, SE = 5.92 × 10^–1^), weighted median (beta = − 7.29 × 10^–1^, SE = 6.22 × 10^–1^); however, the sensitivity analyses results were not statistically significant (all p > 0.05). In addition, genetically predicted maternal smoking and breastfeeding were not causally associated with amblyopia risk using the four distinct MR methods (Table 2).

**Table 2:** Summary results of association for maternal smoking and breastfeeding on amblyopia risk in the MR analysis.

Exposure	Method	Number of SNP	Beta	SE	p value
Maternal smoking	IVW (random effect)	39	− 0.039	0.272	0.886
	IVW (fixed effect)	39	− 0.039	0.272	0.885
	Simple median	39	− 0.248	0.383	0.517
	Weighted median	39	− 0.253	0.372	0.496
Breast feeding	IVW (random effect)	11	− 0.037	0.321	0.907
	IVW (fixed effect)	11	− 0.037	0.513	0.941
	Simple median	11	0.056	0.513	0.937
	Weighted median	11	0.054	0.692	0.937

*SNP* single nucleotide polymorphism, *MR* Mendelian randomization, *IVW* inverse-variance weighted, *SE* standard error.

Horizontal pleiotropy was not observed in the MR Egger regression for BW on amblyopia risk (p = 0.495). No significant heterogeneity for BW on amblyopia was found in the Cochran’s *Q* test, using MR Egger and IVW methods (p of MR-Egger: 0.998, p of IVW: 0.998). An F-statistic of each IV to indicate instrument strength was presented in Supplementary Table 1. The statistical power of the current study was 94%.

## Discussion

The present study assessed the causal relationship between perinatal factors, such as BW, maternal smoking during pregnancy, and breastfeeding after birth on amblyopia using a one-sample MR method. All the perinatal factors have been reported as possible risk factors for amblyopia. A single-sample used for the MR approach included the UKBB data for exposure and outcome factors. The MR results showed that only BW was causally related to amblyopia in contrast with maternal smoking during pregnancy and breastfeeding were not. Additionally, a weak causal relationship was found only through the IVW method. However, a similar trend was observed for the other methods with no pleiotropy or heterogeneity, which could have caused a bias. Our results are consistent with those of previous clinical studies, which suggest that BW is inversely related to amblyopia risk while only a few clinical studies with large sample sizes have elucidated the associations between various perinatal factors and amblyopia risk. In a population-based cohort from the UK ALSPAC study, maternal smoking in the first trimester significantly increased the amblyopia risk by 1.64 times. However, prematurity did not increase the risk of amblyopia^7^. In the Sydney Pediatric Eye Disease Study of 2461 children between 6 and 72 months of age, Amy et al. reported no significant associations between amblyopia and low BW, preterm birth, maternal smoking, or ethnicity^8^. Whereas, in another Australian study with a representative sample of 6-year-old children, low BW, preterm birth, and history of neonatal intensive care unit (NICU) admission drastically increased the risk of amblyopia, in contrast with maternal smoking and breastfeeding. Based on previous studies, the effect of non-ocular perinatal factors on amblyopia risk is controversial. However, the studies were limited by a few confounding factors. Socio-environmental factors, such as whether amblyopia was treated during diagnosis in young children, screening rate according to social class and race, or follow-up loss during the study, could potentially interfere with amblyopia identification. Most previous studies mainly focused on ocular risk factors such as strabismus and refractive error, and non-ocular risk factors were not thoroughly dealt with in-depth. Therefore, there is a need for an objective and fundamental study given the importance of genome research.

In a previous GWAS study, Shaaban et al. identified genetic variants conferring susceptibility to esotropia, suggesting a parent-of-origin effect^9^. Esotropia was said to be inherited as a complex trait by the GWAS. However, no study has demonstrated the causality of any other factor in the development of amblyopia based on genetic inference. Therefore, we assessed the non-ocular risk factors using the well-established MR method, which is an objective analytical method that demonstrates significant causal inferences with amblyopia.

Along with its various advantages, the MR method has been widely used to estimate putative causal relationships between modifiable risk factors and diseases. Genetic variants were used in natural experiments and randomly allocated at conception. Genetic variants are not influenced by behavioral or environmental factors and are far less likely to be affected by bias from confounding and reverse causation. Additionally, the effects are equivalent to lifetime differences, reducing issues related to transient fluctuations in exposures^10^. However, similar to other analytical methods, the MR depends on assumptions; thus, their plausibility should be estimated. Herein, based on previously published results, we established assumptions between the factors and disease. We also found that the relevance of the results between the MR method and clinical studies is plausible and reasonable. Furthermore, because there is a large restriction to collecting genetic data through blood sampling from children with amblyopia, obtaining the UKBB genetic data with a large sample size is efficient and meaningful.

It is noteworthy that BW may be directly related to amblyopia and negatively correlated with amblyopia risk. It has been reported that the lower the BW, the higher the correlation with retinal and neurodevelopmental problems, such as retinopathy of prematurity (ROP), intraventricular hemorrhage, or periventricular leukemia^11–13^. The possibility of secondary visual disorders following these problems can be considered; however, it is necessary to consider that patients with low BW do not necessarily have these diseases. Hou et al. demonstrated that infants with a low BW, in the absence of identifiable retinal or neurologic abnormalities, had a significant effect on visual cortical sensitivity which is an important factor in visual development^14^. Thus, we first attempted to remove all possible SNPs related to any visual or neurologic diseases through a GWAS source to avoid any confounding effect during the analysis. Rs9895335 and rs4977838 were related to cognitive ability and neurological diseases, contributing to the occurrence of amblyopia by influencing brain function and independently affecting the outcome without going through a risk factor. Therefore, we excluded these SNPs from the final analysis, and then finally identified genetic variants related to BW conferring susceptibility to amblyopia, suggesting a parent-of-origin effect. Therefore, BW may have a stronger primary association with amblyopia.

The results of the genetic causality analysis in the current, study may have important clinical implications. It is well known that regular observation for visual development is usually suggested for premature birth with ROP cases. Our results can also suggest the need for regular monitoring of visual development in infants with low BW and a normal gestation period (even without ROP or other neurodevelopmental diseases) to monitor amblyopia risk. This may suggest the potential of BW as a possible predictive factor for visual maldevelopment and the importance of regular follow-up and early intervention in detecting and managing amblyopia.

This study has several limitations. First, owing to the use of summary data, any potential non-linear relationships or stratification effects could not be explored. Second, our findings may not be generalizable to other ethnic groups. Thus, caution is needed to validate the results from MR studies based on three assumptions that should be carefully checked and interpreted in the context of prior biological information. Second, MR is a popular method for estimating the causal effects of risk factors in observational studies; however, it is difficult to completely exclude the effect of relevant unobserved potential confounders. Finally, we could find statistically significant relationship between BW and amblyopia only in IVW model. Further study is required to verify it.

It is the first study to focus on perinatal factors that are difficult to collect from a genetic perspective in clinical studies. We used the MR approach, which can investigate causal effects and largely avoid problems with other observational studies^15^. Additionally, the 10E−06 criterion was established to include a novel gene called "Unknown" in its function from the gene related to BW. Owing to the insufficiency of GWAS amblyopia research, it is necessary to further reinforce the functions of unknown genes and generate relevant SNPs that can be sufficiently validated in future studies.

In conclusion, we found a causal relationship between BW and amblyopia risk in the European population. The results of genetic association may substantially contribute to a possible evidence of the parent-of-origin effect on amblyopia risk and suggest the clinical need to carefully screen for possible amblyopia in low BW infants.

## Materials and methods

### Data source and study population

UKBB is a prospective cohort study of over 500,000 individuals aged between 40 and 69 years across the United Kingdom from 2006 to 2010^16^. Information on blood, urine and saliva samples, physical measurements, and individual answers to an extensive questionnaire focusing on health and lifestyle, were collected. The full data release included a cohort of successfully genotyped samples. Details of the study population and quality control have been described earlier^17^. The study was approved by the North west Multi-Centre Research Ethics Committee and the National Information Governance Board for Health and Social Care (11/NW/0382).

Genetic associations with perinatal factors (BW, maternal smoking, and breast feeding) and amblyopia were obtained from the UKBB pan-ancestry summary statistics (https://pan.ukbb.broadinstitute.org/, released June 16, 2020, assessed January 15, 2021) encompassing people of European ancestry only^16^. GWAS for BW were conducted on approximately 262,966 participants. The individuals evaluated in the GWAS for maternal smoking and breastfeeding consisted of 122,201 and 267,893 cases and 249,727 and 92,087 controls, respectively. GWAS for amblyopia was performed on 417,030 participants comprising 862 cases and 416,168 controls.

### Assumption of Mendelian randomization analysis

**Figure 3:**
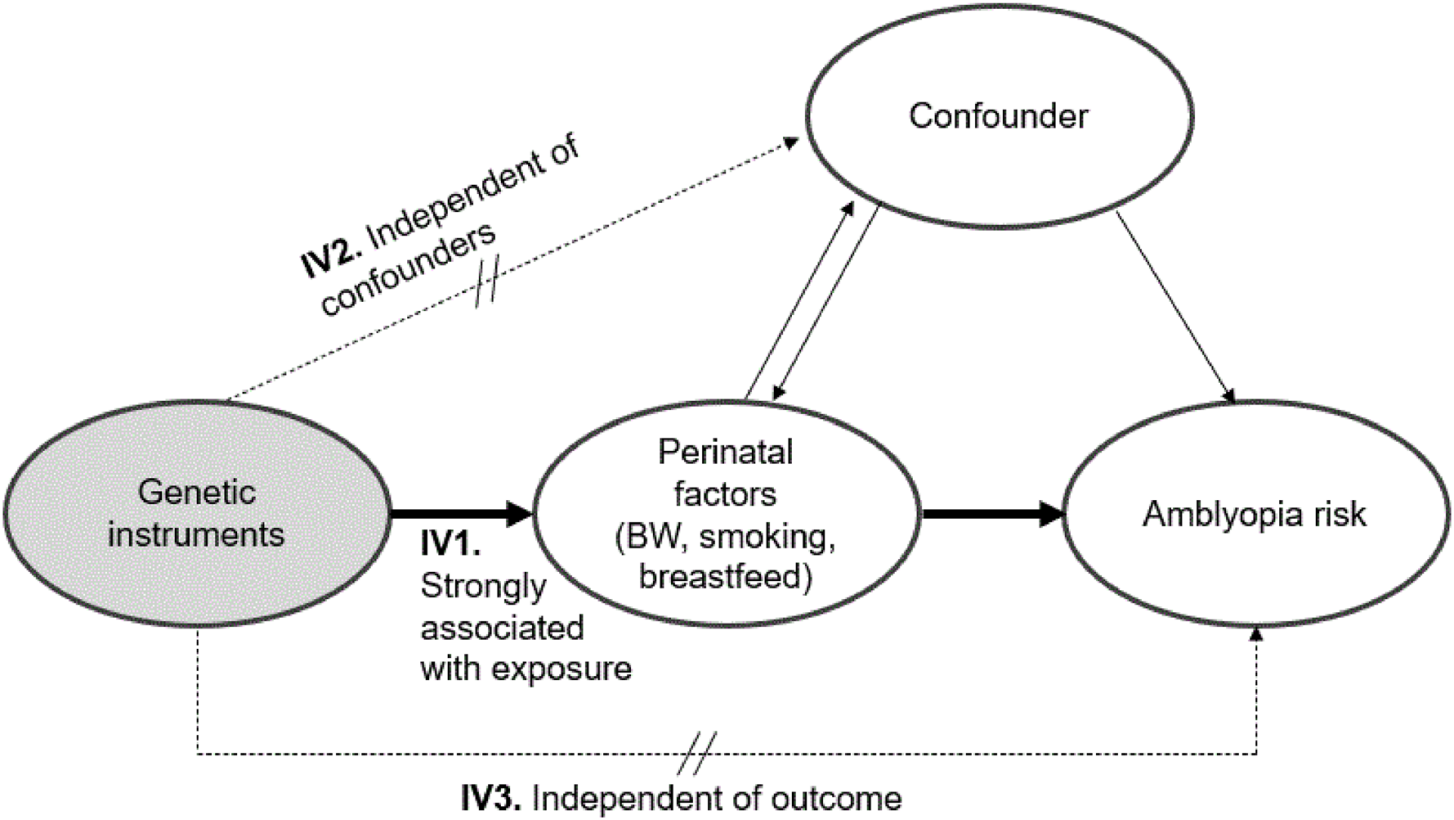
Single-sample Mendelian randomization testing the causal effect of perinatal factors on the risk of developing amblyopia. Estimates of the single nucleotide polymorphism (SNP)–perinatal factor association (instrumental variable [IV]1), and SNP-amblyopia association (IV3) were calculated. Finally, SNP estimates were combined using the MR approach to confirm the overall causal estimate of perinatal factors on amblyopia risk. *BW* birth weight.

We implemented MR analysis in compliance with the STROBE-MR guidelines and the guidelines for performing MR. An MR causal diagram comprised genetic IVs, perinatal factors (exposure), and amblyopia risk (outcomes, Fig. 3). Three assumptions were considered: (1) genetic IVs are strongly associated with perinatal factors, (2) genetic IVs are associated with amblyopia risk only through perinatal factors, and (3) there is an association between genetic IVs and perinatal factors, and amblyopia risk, is unconfounded. Given that only assumption 1 is empirically verifiable, careful consideration of potential violations of assumption 2 (due to linkage disequilibrium (LD), canalization, or horizontal pleiotropy) is important to minimize bias^18^. A SNP that violates these assumptions is referred to as an invalid IV, and its inclusion in MR analyses may bias the results^19^.

### Exposure and outcome

SNPs associated with perinatal factors (BW, maternal smoking, and breastfeeding) at genome-wide significance were identified using IVs, and genetic associations with amblyopia were obtained from summary statistics (beta coefficients and standard errors) provided by UKBB with adjustment for sex, age, and the 20 principal components (available at https://pan.ukbb.broadinstitute.org/downloads/index.html). Pleiotropy is a gene expression in which one gene influences two or more seemingly unrelated phenotypic traits. The location of such a specific gene, which reveals multiple phenotypic expressions, is called a pleiotropic locus. Because it may affect several traits simultaneously, SNPs that could be pleiotropic loci were excluded based on results of phenotype mapping and expression quantitative trait loci (eQTLs) to avoid violating the MR assumptions. We also selected SNPs strongly associated with each perinatal factor to meet the MR assumptions of IVs. To hold the assumption of MR that the genetic variant affects the outcome only through the risk factor, we excluded all possible SNPs likely to be directly related to the outcome. In the final analysis, data not removing SNPs were also estimated in consideration of downstream effect.

### Data harmonization

SNPs associated with each perinatal factor were excluded because of LD clumping (R^2^ ≥ 0.001 within a clumping distance of 10,000 kb) and were palindromic with intermediate allele frequencies. We harmonized the data by aligning all reference alleles in agreement with the effect allele and effect allele frequencies for the palindromic SNPs. Finally, three datasets were created: (1) harmonized data for SNP-birth weight and SNP-amblyopia; (2) harmonized data for SNP-maternal smoking during pregnancy, and SNP-amblyopia; (3) harmonized data for SNP-breastfeeding after birth and SNP-amblyopia.

### Statistical and sensitivity analysis

The causal estimate was calculated in several ways, each of which had different assumptions, and provided the ability to test MR estimate validity. The main causal effect was estimated using the beta coefficients ratio of SNP-amblyopia risk factors to SNP-each perinatal factor, and this was combined across all genetic IVs using the IVW method with a fixed effect/random-effect model, and simple and weighted median regression methods^20,21^.

#### Inverse-variance weighting (IVW) method

This is a method of aggregating two or more random variables to minimize the variance of the weighted average. It is a weighted average of the causal effects of genetic variants^20^. The IVW is optimal because the IVW estimator achieves minimum variance and is equivalent to the two-stage least squares method with summary data. It is biased when at least one genetic variant is invalid in the fixed effects model. The random effect model is analyzed assuming there are invalid IVs.

#### Simple median estimator

It calculates the median of the ratio IV estimates evaluated using each genetic variant individually and has greater robustness with strongly outlying causal estimates compared with MR-Egger methods. In addition, it provides a consistent estimate of the causal effect when at least 50% of the genetic variants are valid IV. However, it is inefficient compared to the IVW and weighted median methods.

#### Weighted median estimator

This method is similar to the simple median method. A causal effect can be calculated as the median of the weighted ratio estimates using the reciprocal of the variance of the ratio estimate as weights when at least 50% of the weight originates from valid IVs^21^. In contrast to the MR-Egger process, it has the benefit of preserving greater precision in estimates. The efficiency of this method was similar to that of the IVW method.

### Power analysis and availability of data

We estimated statistical power for MR using a web-based calculator (https://shiny.cnsgenomics.com/mRnd/), by assuming that the significance level α of 0.05 and the r-square of 0.053. Given those parameters, statistical power was calculated. Summary statistics from genome-wide association studies for BW, breast feeding, maternal smoking, and amblyopia in UKBB data are publicly available online: http://www.nealelab.is/uk-biobank.

### Ethical statement

This study relied on de-identified summary-level data that have been made publicly available on patient-level UKBB phenotypic and genetic data. Institutional Review Board/Ethics Committee approval from Seoul National University in Seoul, South Korea was obtained for the UKBB data usage.

## Supplementary Information

Supplementary Figure 1.

Supplementary Figure 2.

Supplementary Table 1.

Supplementary Table 2.

Supplementary Table 3.

Supplementary Table 4.
