# Supplementary figures and images for: Genetic causal inference between amblyopia and perinatal factors
Source: Sci Rep. 2022 Oct 27;12:18050. doi: 10.1038/s41598-022-22121-3 (PMC9613760; doi:10.1038/s41598-022-22121-3)

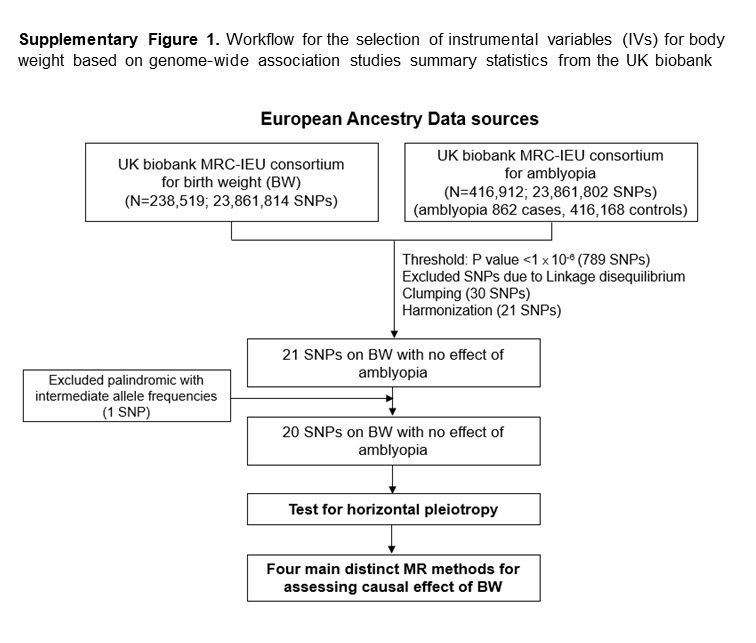

Supplement: Supplementary file 1 — Supplementary Figure 1. [file 41598_2022_22121_MOESM1_ESM.tif]

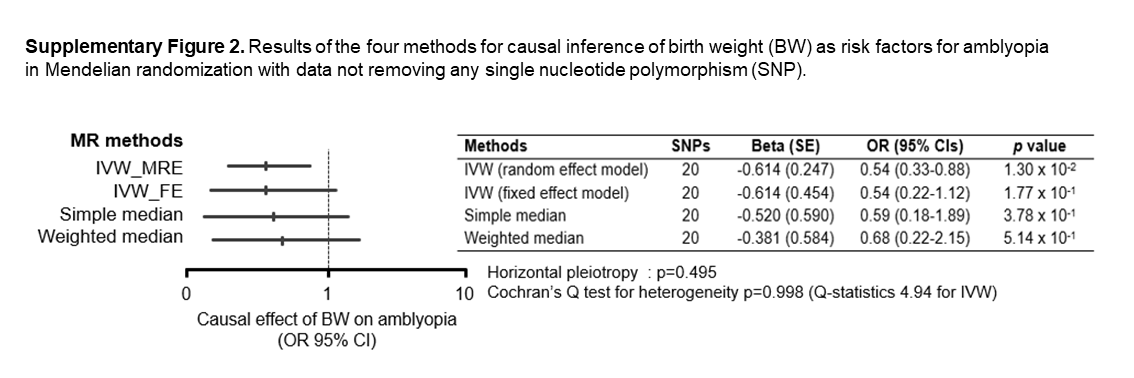

Supplement: Supplementary file 2 — Supplementary Figure 2. [file 41598_2022_22121_MOESM2_ESM.tif]
