# Supplementary material for: Genetic causal inference between amblyopia and perinatal factors
Source: Sci Rep. 2022 Oct 27;12:18050. doi: 10.1038/s41598-022-22121-3 (PMC9613760; doi:10.1038/s41598-022-22121-3)
Supplement: Supplementary file 3 — Supplementary Table 1. [file 41598_2022_22121_MOESM3_ESM.docx]

**Supplementary Table 1.** Summary statistics of Single nucleotide polymorphisms (SNPs) associated with birth weight and those of SNPs for amblyopia from the results of genome-wide association study in UK biobank European ancestry

| **SNP** | **Function** | **Gene** |  | **Birth weight** | | |  | **Amblyopia** | | |
| --- | --- | --- | --- | --- | --- | --- | --- | --- | --- | --- |
|  |  |  | **Alleles** | **EAF** | **F-statistics** | **Beta (SE)** | **p-value** | **EAF** | **Beta (SE)** | **p-value** |
| rs74226445 | intergenic | ZBTB18;C1orf100 | G/C | 0.015 | 35.44 | /0.121 (0.020) | 2.63E-09 | 0.015 | -0.011 (0.220) | 0.96 |
| rs151320013 | intergenic | NRP1;LINC00838 | G/A | 0.022 | 31.94 | /0.096 (0.017) | 1.60E-08 | 0.021 | -0.020 (0.182) | 0.91 |
| rs116552258 | ncRNA_intronic | LINC00461 | C/T | 0.113 | 31.31 | /0.042 (0.008) | 2.19E-08 | 0.104 | -0.084 (0.082) | 0.30 |
| rs72930125 | intergenic | LINC01919;MBD2 | G/A | 0.061 | 30.92 | -0.055 (0.01) | 2.69E-08 | 0.058 | -0.063 (0.107) | 0.56 |
| rs8022105 | intergenic | LOC105370473;LINC02302 | C/T | 0.535 | 30.15 | /0.026 (0.005) | 3.99E-08 | 0.535 | /0.014 (0.051) | 0.79 |
| rs4977838 | intergenic | LINC01239;LOC101929563 | T/G | 0.436 | 29.36 | /0.026 (0.005) | 6.00E-08 | 0.434 | -0.003 (0.052) | 0.96 |
| rs3906525 | intergenic | KCTD16;PRELID2 | G/T | 0.125 | 29.04 | /0.039 (0.007) | 7.08E-08 | 0.121 | -0.027 (0.079) | 0.73 |
| rs56031201 | UTR3 | PDGFB | G/C | 0.019 | 28.86 | /0.096 (0.018) | 7.84E-08 | 0.015 | -0.239 (0.193) | 0.22 |
| rs9895335 | intronic | TOM1L2 | A/G | 0.655 | 28.70 | /0.027 (0.005) | 8.38E-08 | 0.658 | /0.025 (0.054) | 0.65 |
| rs1074078 | intergenic | MTOR;UBIAD1 | C/T | 0.330 | 28.06 | -0.027 (0.005) | 1.17E-07 | 0.340 | /0.037 (0.054) | 0.50 |
| rs1385865 | intronic | KCNH7 | C/T | 0.504 | 28.04 | -0.025 (0.005) | 1.19E-07 | 0.517 | /0.043 (0.051) | 0.41 |
| rs3746038 | UTR3 | KLF16 | C/T | 0.201 | 26.83 | -0.031 (0.006) | 2.23E-07 | 0.208 | /0.029 (0.064) | 0.65 |
| rs35991747 | intronic | SCTR | A/G | 0.508 | 26.40 | /0.025 (0.005) | 2.79E-07 | 0.494 | -0.046 (0.053) | 0.38 |
| rs111654718 | intergenic | SNX16;LOC101927141 | G/A | 0.007 | 26.30 | -0.146 (0.028) | 2.94E-07 | 0.007 | -0.153 (0.303) | 0.61 |
| rs10202061 | intergenic | KCNJ3;LINC01876 | G/A | 0.185 | 25.99 | /0.031 (0.006) | 3.43E-07 | 0.174 | -0.068 (0.066) | 0.30 |
| rs7218341 | intergenic | LOC105371910;LINC01993 | C/G | 0.104 | 25.73 | /0.041 (0.008) | 3.91E-07 | 0.104 | /0.011 (0.087) | 0.90 |
| rs1348694 | intergenic | LINC02220;DNAH5 | T/C | 0.420 | 24.79 | -0.024 (0.005) | 6.40E-07 | 0.427 | /0.022 (0.052) | 0.68 |
| rs6952555 | intergenic | CTTNBP2;LSM8 | T/C | 0.319 | 24.49 | -0.025 (0.005) | 7.49E-07 | 0.322 | /0.009 (0.055) | 0.87 |
| rs9283778 | intergenic | LINC02239;LINC02228 | G/T | 0.774 | 24.32 | /0.028 (0.006) | 8.20E-07 | 0.766 | -0.040 (0.061) | 0.52 |
| rs2281228 | intronic | IFT140;TMEM204 | C/T | 0.176 | 24.00 | -0.030 (0.006) | 9.57E-07 | 0.179 | /0.010 (0.067) | 0.88 |

UKBB; UK biobank, SNP, Single nucleotide polymorphism; GWAS, genome-wide association study; EAF, effect allele frequency; SE, standard error.
