# Supplementary material for: Genetic causal inference between amblyopia and perinatal factors
Source: Sci Rep. 2022 Oct 27;12:18050. doi: 10.1038/s41598-022-22121-3 (PMC9613760; doi:10.1038/s41598-022-22121-3)
Supplement: Supplementary file 4 — Supplementary Table 2. [file 41598_2022_22121_MOESM4_ESM.docx]

**Supplementary Table 4.** Summary results of association for birthweight on amblyopia risk in the MR analysis according to the different level of significance level.

| **Methods** | **SNP** | **Beta (SE)** | **OR (95% CIs)** | ***p* value** | **Comment** |
| --- | --- | --- | --- | --- | --- |
| ***Significance level of 1E-07*** |  |  |  |  | 1) The number of IVs at each level was insufficient. It may affect the implementation of sensitivity analysis such as MR-Egger and make the estimation of pleiotropic variance inaccurate.^1^ Here, the result from MR bootstrap was reversed compared to the result from MR-Egger, because the number of IVs was small.  2) If there are many SNPs with estimate values of possible invalid IVs, the beta value is known to be unstable in the mode-based analysis.^2^ The estimates may become unstable in MR-Egger and mode-based analysis.^3^  3) Even when all SNPs are valid IVs, the combined explanatory power of SNPs is still small, then weak instrument bias may occur when a small number of IVs are used for analysis. |
| IVW (random effects) | 9 | -0.323 (0.404) | 0.72 (0.33-1.60) | 0.424 |  |
| IVW (fixed effects) | 9 | -0.323 (0.649) | 0.72 (0.20-2.58) | 0.618 |  |
| Simple median | 9 | //-0.104 (0.836) | 0.90 (0.18-4.64) | 0.901 |  |
| Weighted median | 9 | -0.102 (0.849) | 0.90 (0.17-4.76) | 0.904 |  |
| MR Egger | 9 | -1.127 (1.419) | 0.32 (0.02-5.22) | 0.456 |  |
| MR Egger (bootstrap) | 9 | 0.657 (1.616) | 1.93 (0.08-45.84) | 0.336 |  |
| Simple mode | 9 | -0.016 (1.292) | 0.99 (0.08-12.39) | 0.991 |  |
| Weighted mode | 9 | -0.029 (1.256) | 0.97 (0.08-11.39) | 0.982 |  |
| ***Significance level of 5E-08*** |  |  |  |  |  |
| IVW (random effects) | 5 | -0.131 (0.516) | 0.87 (0.32-2.41) | 0.800 |  |
| IVW (fixed effects) | 5 | -0.131 (0.852) | 0.87 (0.17-4.66) | 0.878 |  |
| Simple median | 5 | -0.096 (1.086) | 0.92 (0.11-7.72) | 0.936 |  |
| Weighted median | 5 | -0.091 (1.037) | 0.91 (0.12-6.99) | 0.931 |  |
| MR Egger | 5 | -0.222 (1.755) | 0.80 (0.03-24.99) | 0.907 |  |
| MR Egger (bootstrap) | 5 | /0.052 (2.523) | 1.05 (0.01-147.96) | 0.494 |  |
| Simple mode | 5 | 0.174 (1.368) | 1.19 (0.08-17.39) | 0.905 |  |
| Weighted mode | 5 | 0.135 (1.384) | 1.14 (0.08-17.24) | 0.927 |  |

Reference: 1. Systematic review of Mendelian randomization studies on risk of cancer, 2. Profile-likelihood Bayesian model averaging for two-sample summary data Mendelian randomization in the presence of horizontal pleiotropy, 3. Robust inference in two-sample Mendelian randomization via the zero modal pleiotropy assumption
