# Supplementary material for: Genetic causal inference between amblyopia and perinatal factors
Source: Sci Rep. 2022 Oct 27;12:18050. doi: 10.1038/s41598-022-22121-3 (PMC9613760; doi:10.1038/s41598-022-22121-3)
Supplement: Supplementary file 5 — Supplementary Table 3. [file 41598_2022_22121_MOESM5_ESM.docx]

**Supplementary Table 3.** Summary statistics of Single nucleotide polymorphisms (SNPs) associated with breast feeding and those of SNPs for amblyopia from the results of genome-wide association study in UK biobank European ancestry.

| **SNP** | **Function** | **Gene** |  | **Breast feeding** | | | **Amblyopia** | | |
| --- | --- | --- | --- | --- | --- | --- | --- | --- | --- |
|  |  |  | **Alleles** | **EAF** | **Beta (SE)** | **p-value** | **EAF** | **Beta (SE)** | **p-value** |
| rs4575516 | intergenic | RBFOX1;TMEM114 | A/G | 0.309 | /0.034 (0.006) | 6.71E-08 | 0.304 | -0.008 (0.056) | 0.89 |
| rs533821858 | UTR3 | MPP6 | A/G | 0.155 | /0.044 (0.008) | 1.19E-07 | 0.144 | -0.083 (0.073) | 0.26 |
| rs4731905 | intronic | CHCHD3 | C/A | 0.251 | -0.035 (0.007) | 1.42E-07 | 0.245 | -0.043 (0.059) | 0.47 |
| rs2837998 | intronic | BACE2 | G/A | 0.602 | -0.031 (0.006) | 2.24E-07 | 0.602 | -0.013 (0.052) | 0.81 |
| rs62370518 | intergenic | FST;NDUFS4 | A/G | 0.164 | -0.041 (0.008) | 2.46E-07 | 0.171 | /0.038 (0.070) | 0.58 |
| rs150789461 | UTR5 | DVL1 | G/A | 0.928 | -0.059 (0.012) | 3.13E-07 | 0.926 | -0.048 (0.101) | 0.64 |
| rs3767657 | intronic | EPRS1 | C/T | 0.130 | /0.045 (0.009) | 3.61E-07 | 0.135 | /0.056 (0.078) | 0.47 |
| rs4799518 | ncRNA_intronic | LINC01902 | C/T | 0.192 | /0.038 (0.007) | 3.76E-07 | 0.190 | /0.002 (0.066) | 0.97 |
| rs62422906 | intergenic | TBX18;LINC02535 | C/G | 0.114 | /0.047 (0.009) | 3.80E-07 | 0.117 | /0.045 (0.081) | 0.58 |
| rs2442723 | intergenic | LINC02571;HLA-B | G/A | 0.199 | -0.038 (0.008) | 5.87E-07 | 0.207 | /0.043 (0.066) | 0.52 |
| rs2238646 | intronic | PDE4C | G/A | 0.081 | /0.053 (0.011) | 7.91E-07 | 0.077 | -0.048 (0.094) | 0.61 |

UKBB; UK biobank, SNP, Single nucleotide polymorphism; GWAS, genome-wide association study; EAF, effect allele frequency; SE, standard error.
